# Survey on public awareness, attitudes and self-protective behavior adoption in different periods of COVID-19

**DOI:** 10.3389/fpubh.2022.1063384

**Published:** 2023-01-20

**Authors:** Fang Fang, Sijie Chen, Xianhui Geng, Emmanuel Kiprop

**Affiliations:** ^1^School of Economics and Humanities, Jiangsu Vocational College of Agriculture and Forestry, Jurong, China; ^2^College of Economics and Management, Nanjing Agricultural University, Nanjing, China; ^3^School of Business and Economics, Kabarak University, Nakuru, Kenya

**Keywords:** COVID-19, KAP, PADM, behavior adoption, different periods

## Abstract

**Introduction:**

The outbreak and spread of the pandemics have been an issue of critical concern globally, posing a significant threat to the health sector globally. This study aimed to examine the basic knowledge and attitudes toward the recommended protective measures at different times, respond to the COVID-19 pandemic, and provide recommendations for developing targeted strategies and measures for preventing and controlling public health emergencies.

**Methods:**

The study used self-filled questionnaires to examine the public's knowledge, attitudes, and practices on COVID-19 at two different period, from 20 to 31 March 2020 (the beginning period) and 22–27 April 2022 (the regular epidemic prevention and control period). Descriptive and quantitative analyses were used for statistical analysis.

**Results and discussion:**

The survey collected 2375 valid questionnaires. A comparison of the two periods reveals that as the epidemic continued over a long period, the level of knowledge, attitudes toward preventive measures, risk perceptions, and adoption behavior of the respondents at the beginning of the epidemic were significantly higher than during the regular epidemic prevention and control period. With the upsurge in the spread of the epidemic, the public needs a multi-channel, targeted, and all-round guidance and information on prevention and control of the COVID-19, and internalizes knowledge into individual's behavior of actively responding to diseases.When the epidemic lasts for a long time, the relevant agencies should strengthen their monitoring role to promote public compliance with the recommended measures.

## Introduction

Public Health Emergencies are a common threat to human survival. In recent years, public health emergencies have frequently occurred globally with an increasingly expanding scope of influence (1, 2). In early 2020, the COVID-19 virus outbreak poses a severe threat to human life and health due to its rapid, widespread and highly contagious nature. It has posed an unprecedented challenge to the global public health system and government governance capacity (3). COVID-19 is the most rapidly spreading, widely infected and difficult to prevent and control major global public health event that has occurred in China.

The alarming incidence of COVID-19 and the resulting mass casualties have severely strained limited healthcare resources. Increasingly advanced technologies are being used to prevent the disease, such as early diagnosis and accurate classification of COVID-19 patients using x-ray images and voice signal processing techniques, the use of large amounts of data to track down people in close contact with infected individuals rapidly, and so on (4, 5).

At the same time, countries have put in place strict measures to prevent and control epidemics. For example, China “closed” high-risk cities at the epidemic's beginning (e.g., Wuhan residents were not allowed to leave the city from 23 January 2020). During the regular epidemic prevention and control period, the Chinese government requires the tracking and isolation of people from high-risk areas in the epidemic, the testing of body temperature when entering public places, the mandatory wearing of masks and the reduction of gatherings. In the United States, “workplace quarantine, temperature testing, and virus testing” and in some states, “14-day home orders” and “no gatherings of more than 10 people” were implemented. The public's active participation and compliance with the relevant systems and regulations are crucial to the prevention and control of the epidemic. However, there are many negative and non-compliant behaviors in the epidemic prevention and control system.

Unfortunately, there are still many people who do not follow these preventive recommendations. On 13 January, 2020, a man in Bengbu concealed a history of close contact with his relatives in Wuhan, leading to the emergency quarantine of 27 health care workers and 61 hospital patients (6). 21 July 2021, a woman in Yangzhou concealed a trip to Nanjing and frequently moved in crowded places, leading to the outbreak's spread in Yangzhou (7). These negative behaviors all reflect the non-compliance characteristics of the public toward the epidemic prevention and control measures, leading to the spread of the epidemic and posing a significant threat to the lives and health of the general public (8). At the same time, with the mutation of COVID-19, especially the emergence of the new Omicron, the current COVID-19 outbreak in China is still frequently occurring in different regions and spreading widely. Therefore, it is of great practical importance to discuss how to promote public compliance in the context of epidemic prevention and control. Although humans have defeated many past pandemics, future pandemics are unpredictable and inevitable. Hence, it is highly significant to develop public health solutions for pandemic prevention and control.

Most previous research on public health emergencies has focused on the epidemic's peak. However, as the epidemic situation changes, the public's focus varies during each period, leading to different compliance behaviors. There is a lack of research on public adoption of preventive measures at the regular epidemic prevention and control period. It is important to understand the behavior of the public during routine outbreak prevention and control when the epidemic lasts for a longer time. At the same time, comparative analyses for different periods of the same public health emergency are mostly conducted using retrospective surveys, and respondents may suffer from memory bias. Therefore, a questionnaire was creatively designed for this study to be administered during the peak and normal periods of the outbreak, effectively reducing the memory bias of the interviewees.

In the present study, we aimed to describe the dynamics of public awareness, attitudes, and adoption of self-protective behaviors among the Chinese population during the COVID-19 outbreak and during the regular epidemic prevention and control period, and to explore the reasons affecting adoption behaviors at different times. Findings from the study are expected to provide essential policy recommendations to the Public health department to help in decision-making, especially those related to epidemic prevention and disease control.

The Knowledge, Attitude, Practice model (KAP) and the Protective Action Decision Model (PADM) are frameworks for understanding public compliance with prevention recommendations to reduce the spread of epidemics (9, 10). The Knowledge, Attitude, Practice model is one of the models aimed at changing human health-related behavior. It is a behavior intervention theory, dividing the change in human behavior into three continuous processes of acquiring knowledge, conceptualizing ideas and forming responses.

Knowledge is the basis of action. The paradigm of disease prevention and health promotion depends mainly on understanding health behavior (11). When the public thinks that the disease will seriously affect health, they will choose to implement preventive and protective measures (12, 13). The lack of relevant knowledge was a significant reason for the epidemic rampage (14, 15). This idea guides the development of the hypothesis of this study, as follows:

H1: The degree of relevant knowledge is positively correlated with the intention of adoption.

Attitude change is the key to behavior adoption, and attitude is the driving force for behavioral change. Only when people form corresponding beliefs is when they can adopt a positive attitude to change their behavior. The more correct attitudes are, the higher the coordination of public actions (16).

From this we can hypothesize that:

H2: Attitude is positively related to the intention of adoption.

In the context of a rapidly developing media industry, rumors often emerge during diseases. The efficiency of information dissemination has a lasting impact on the prevention and control of an epidemic. Understanding the sources of public information about infectious diseases and the media channels they prefer to obtain information can provide the government with valuable information. Inaccurate health information can mislead the public and hinder the implementation of more effective measures (17). From this, we can hypothesize that:

H3: The information resolution ability is positively related to the adoption intention.

The Protective Action Decision Model (PADM) combines individuals' social environment, relevant information obtained, and the relevant personal risk experience. PADM contains three kinds of perception: risk perception, protective action perception, and stakeholder perception. It provides suggestions for disaster reduction by investigating the public's perception of these three aspects (18).

Empirically, an individual's subjective assessment of risk has been described as “risk perception” which describes the degree of the expected impact of a person exposed to potential risk. Specifically, the experiences of family members and friends around us in disasters are also included in personal risk experiences and information obtained from government authorities or media can improve risk perception (19). Risk perception forms the fundamental determinant of people's prevention of life-threatening events (20, 21). The lack of relevant information has caused the public misinformation on the viral disease currently experienced. Existing research shows that people who have a higher awareness of risk tend to have stronger willingness to adopt protective actions (22, 23). It can therefore be assumed that:

H4: Risk perception is positively related to the adopted intention.

There are two fundamental attributes of protective action perception, namely hazard-related attributes and resource-related attributes (24). Hazard-related attributes reflect the relationship between risk and protective behavior and reflect an individual's perception of the ability of the recommended preventive and protective measures to reduce risk. When people have a higher level of perception of hazard-related attributes, they tend to be more confident on the possibility of taking protective actions to reduce risks, and the actual adoption of behaviors will increase (25, 26).

Unlike the hazard-related attributes that emphasize the protective action on the risk, resource-related attributes measure the cost of adopting protective actions, including time, money, and the degree of cooperation required, and reflect the relationship between the resources spent and the protective actions. During the outbreak of infectious diseases, individuals will encounter obstacles such as forgetting to wash their hands, insufficiency of space to maintain social distancing, bearing the risk of vaccine side effects among others. When people perceive a higher level of resource demand, they will reduce their confidence in risk adjustment. The perceived high level of resource demand often leads to a low level of prevention behavior adoption. From this we can hypothesize that:

H5: The perception of hazard-related attributes is positively correlated to adopt preventive measures.
H6: Resource-related attribute perception is inversely related to the intention to adopt preventive measures.

Stakeholder perception is an individual's view of stakeholders' expertise, credibility, and protection responsibilities. People's distrust of the government and experts exacerbated existing concerns on preventive measures' effectiveness (27–29). Lack of trust in the governmental institutions and experts may overestimate the development of the epidemic, thereby being too nervous and causing panic (8, 27, 30). Strengthening the communication between the government and the public is conducive to enhancing public trust in the government (31), ensuring the public's correct understanding of the epidemic (32). It can therefore be assumed that:

H7: The public's perception of stakeholders is positively correlated with the adopted intentions.

**Figure 1:**
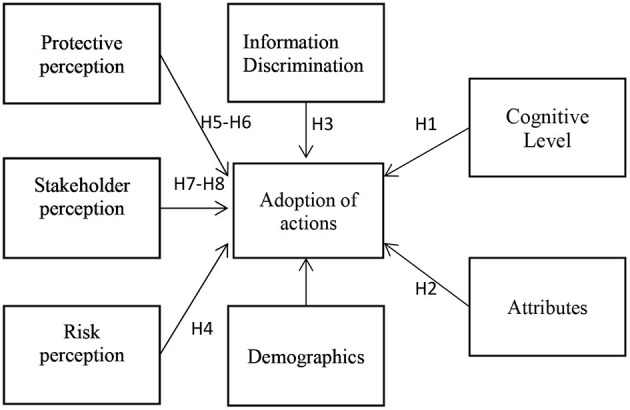
Framework diagram.

Knowledge is the basis of action. Attitude change is the key to behavior adoption, and attitude is the driving force for behavioral change. Risk perception, protective action perception, and stakeholder perception are correlated with the adopted intentions. Based on the previous literature review and hypothesis proposal, the proposed operational mode is depicted in Figure 1.

## Methodology and data

### Research instrument

We chose to distribute the questionnaires through an online platform to collect the data. The first cross-sectional survey on the status of the COVID-19 in China was conducted from 10 to 20 March 2020 for the “beginning period.” The second cross-sectional survey, entitled “regular epidemic prevention and control period,” was conducted from 22 to 27 April 2022. Although it was not possible to conduct a national community-based sample during that time, the data was collected electronically using Wenjuanxing.

Before the survey, participants were informed of the purpose of the study, assured of personal information confidentiality, and informed of their right to participate voluntarily. Participants were deemed to have given informed consent by beginning to complete the questionnaire after carefully reading the instructions section. This study resulted in a valid sample of 2,315 questionnaires from 30 provinces, municipalities and autonomous regions in China, excluding Tibet, Taiwan, Hong Kong and Macao, by removing those questionnaires that took <4 min to answer and those with 20 consecutive identical answers.

### Measurement of key variables

The questionnaire consists of four parts. The first part deals with collecting general demographic data, such as gender, age, education and location. The second part presents the general knowledge of COVID-19. Five items were designed for the transmission route of COVID-19, susceptible groups, symptoms of infection, preventive measures and days of isolation. Participants who selected the three correct options were considered aware of transmission routes. The four options for susceptible populations were: generally susceptible, young people are not susceptible, people who smoke and drink regularly are not susceptible, and people who have exposure to the virus are susceptible. The options for symptoms of infection are “fever, dry cough”; “nasal congestion, cough”; “weakness, shortness of breath,” “diarrhea.” The researchers hypothesized that if participants selected “fever, dry cough,” “weakness, shortness of breath,” and “diarrhea,” then they had a high awareness of the clinical features. Four options of protection were included, namely “cloth mask,” “activated charcoal mask,” “medical surgical mask,” and “N95 protective mask.” The researchers hypothesized that participants would have a higher awareness of protective measures if they chose the “medical-surgical mask” and “N95 protective mask”. Four options were set for the duration of isolation, and those who chose 14 days were considered to understand better.

The third section includes adopting behaviors to prevent COVID-19, including keeping social distance, reducing travel, actively taking body temperature and wearing a mask. The potential possibility ranges from utterly impossible to affirmative realization, respectively 1 to 5 points. When each preventive measure's score is less than or equal to 3 points, it is regarded as bad behavior.

The fourth section includes respondents' judgments on information screening for COVID-19, attitudes toward preventive behaviors and stakeholder perceptions. The attitudes toward preventive measures in the study included whether they support the preventive measures, whether the preventive measures can effectively reduce exposure to infection and whether a citizen takes the precautionary measures. Express the respondents' views by measuring preventive measures' hazard-related attributes and resource-related attributes. The stakeholders' views were measured from their understanding of the COVID-19 pandemic and their responsibilities. The risk perception focused on the respondents' knowledge of the disease rate and mortality in terms of risk perception. They were all measured using Likert's 5-point scale Where “1” in the range represented utterly disagree, “3” generally was used to show the respondent neither agreed nor disagreed, whereas “5” represented utterly agree, and the average value is used as the measurement (See Table 1).

**Table 1:** Measurement of key variables.

**Variable**	**Question**
Intention to comply with recommended protective actions 1 = not at all likely 5 = almost certain	How likely is it that you would maintain good hygiene and timely disinfection?
	How likely is it that you would reduce going out?
	How likely is it that you would actively follow changes in your body temperature?
	How likely is it that you would wear masks?
Risk perception 1 = not at all likely 5 = almost certain	How likely do you think you are to get COVID-19 if you go out with a mask?
	How contagious do you think the COVID-19 is?
	How likely do you think you are to get COVID-19 if you receive a courier from a region with a severe outbreak?
	How likely do you think it is that you will die from getting COVID-19?
Attributes of recommended protective actions 1 = utterly disagree 5 = utterly agree	You support compliance with the recommended measures.
	Complying with recommended actions can protect your health.
	It is a citizen's duty to take preventive measures.
Stakeholder perception 1 = not at all likely 5 = to a very great extent	To what extent would you think that local community doctors/local city or state hospital doctors/local health department personnel/provincial or national public health department personnel/local government elected officials are knowledgeable about the COVID-19 virus?
	To what extent would you think local community doctors/local city or state hospital doctors/local health department personnel/provincial or national public health department personnel/local government elected officials are responsible for protecting you from the COVID-19 virus?
Information Discrimination 1 = utterly disagree 5 = utterly agree	For unconfirmed articles and information about the COVID-19 on the internet will not affect your normal life
	You think you know a lot about COVID-19
	You can effectively distinguish between rumors
Dangerous attributes 1 = utterly disagree 5 = utterly agree	Adherence to recommended measures can be effective in protecting health
	Complying with the recommended protective actions would also be useful for purposes other than avoiding COVID-19
Resource attributes 1 = utterly disagree 5 = utterly agree	Complying with the recommended protective action would cost a lot of money
	Complying with the recommended protective action would require a lot of effort or time
	Complying with the recommended protective action would require a lot of cooperation from others

### Empirical method

While such descriptive analysis is based on a simple tabulation, it requires multivariate regressions to identify the multiple factors that can jointly determine the public's preventive measures decisions. As the public has five levels of willingness to accept behavior, which are ordered discrete variables, given the nature of the variables and the content of the study, this paper uses a logistic regression model to estimate the probability that a person is likely to adopt. The logistic regression model estimated the probability that a person might adopt, as a function of all factors that could potentially affect the public's decision to adoption; specifically, we will have:


$$\begin{array}{l} \ln\;\frac{p}{1-p}=\partial +\overset{n}{\underset{i=1}{\sum }}\beta_{i}x_{i}+\varepsilon \end{array}$$

P is the probability of person to adopt protective behavior, Xi are the independent variables that are expected to influence P. Y is the number of adoption protective behavior.

## Results

### Data analysis

You may insert up to 5 heading levels into your manuscript as can be seen in “Styles” tab of this template. These formatting styles are meant as a guide, as long as the heading levels are clear, Frontiers style will be applied during typesetting.

#### Sample profile

This summary of responses was obtained from those who participated in the survey during the beginning period (10–20 March 2020) and the regular epidemic prevention and control period (22–27 April 2022). Total responses were a combination of both. The survey collected 2,375 valid questionnaires. Among the 2,375 respondents, women and men accounted for 42.36 and 57.64%, respectively. In terms of education level, 77.68% of the respondents graduated from college/university, which showed that most of the respondents were educated and had a relatively clear understanding of the judgment of related items. In terms of monthly income, 31.71% of respondents had their annual household income ranging between ¥ 90,000–199,999. In terms of living location, only 6.57% of interviewees resided in high-risk areas while 93.43% lived in low-risk areas. Furthermore, 55.49% of the respondents lived in the cities and 97.47% of interviewees didn't have contact with confirmed patients. The specific situation is provided in Table 2.

**Table 2:** Descriptive statistics.

**Variable**	**Descriptive**	**Beginning period, *n* (%)**	**Regular epidemic prevention and control period, *n* (%)**	**Total, *n* (%)**
Gender	Female	760 (59.33)	609 (55.67)	1,369 (57.64)
	Male	521 (40.67)	485 (44.33)	1,006 (42.36)
Education	Junior high school	93 (7.26)	6 (0.55)	99 (4.17)
	High school	193 (15.07)	67 (6.12)	260 (10.95)
	College/university	890 (69.48)	955 (87.29)	1,845 (77.68)
	Master's degree and above	105 (8.20)	66 (6.03)	171 (7.20)
Annual household income	Below ¥30,000	159 (12.41)	50 (4.57)	209 (8.80)
	¥ 30,000–59,999	235 (18.35)	118 (10.79)	353 (14.86)
	¥ 60,000–89,999	246 (19.20)	218 (19.93)	464 (19.54)
	¥ 90,000–1,99,999	346 (27.01)	407 (37.20)	753 (31.71)
	Over ¥ 1,20,000	295 (23.03)	301 (27.51)	596 (25.09)
Regional risk	High-risk areas	86 (6.71)	70 (6.40)	156 (6.57)
	Low-risk areas	1,195 (93.29)	1,024 (93.60)	2,219 (93.43)
Living location	City	731 (57.06)	587 (53.66)	1,318 (55.49)
	Town	258 (20.14)	412 (37.66)	670 (28.21)
	Rural area	292 (22.79)	95 (8.68)	387 (16.29)
Experience	Is a confirmed patient	3 (0.23)	7 (0.64)	10 (0.42)
	Contact with confirmed patients	17 (1.33)	33 (3.02)	50 (2.11)
	No contact with confirmed patients	1,261 (98.44)	1,054 (96.34)	2,315 (97.47)

#### Respondent's overall knowledge level

The research data revealed that the majority of the participants had a high level of knowledge of preventive measures and days of isolation for novel coronary pneumonia, with 73.14 and 97.56% of respondents answering correctly. The lowest correct rate was for symptoms of infection, with only 10.15% of respondents answering correctly.

It can be seen that most residents maintained high levels of prevention, and had the relevant knowledge, especially on the protection and prevention measures, strictly related to life. Nevertheless, they lacked knowledge on more professional approaches to managing the virus, such as disease transmission and symptoms.

The survey time showed a 0.01 level of significance for the overall knowledge level, except for the knowledge of quarantine days. The respondent's level of knowledge at the beginning of the epidemic was significantly higher than during the regular period of the epidemic. The result shows that most residents are more concerned and knowledgeable about the epidemic at the beginning and that prevention and control education is better at the beginning of the epidemic. The specific situation is provided in Table 3.

**Table 3:** The public's knowledge about COVID-19.

		**Beginning period, *n* (%)**	**Regular epidemic prevention and control period, *n* (%)**	**Total**	**χ^2^**	***p***
Disease transmission	Incorrect	998 (77.91)	988 (90.31)	1,986 (83.62)	66.275	0.000^***^
	Correct	283 (22.09)	106 (9.69)	389 (16.38)		
	Total	1,281	1,094	2,375		
People easily infected	Incorrect	686 (53.55)	641 (58.59)	1,327 (55.87)	6.08	0.014^**^
	Correct	595 (46.45)	453 (41.41)	1,048 (44.13)		
	Total	1,281	1,094	2,375		
Infection symptoms	Incorrect	1,086 (84.78)	1,048 (95.80)	2,134 (89.85)	78.56	0.000^***^
	Correct	195 (15.22)	46 (4.20)	241 (10.15)		
	Total	1,281	1,094	2,375		
Protective measures	Incorrect	295 (23.03)	343 (31.35)	638 (26.86)	20.81	0.000^***^
	Correct	986 (76.97)	751 (68.65)	1,737 (73.14)		
	Total	1,281	1,094	2,375		
Quarantine days	Incorrect	25 (1.95)	33 (3.02)	58 (2.44)	2.808	0.094^*^
	Correct	1,256 (98.05)	1,061 (96.98)	2,317 (97.56)		
	Total	1,281	1,094	2,375		

^***^p < 0.001, ^**^p < 0.01, ^*^p < 0.05.

#### Respondent's attitude toward preventive measures

According to the survey data, the respondents expressed support for the preventive measures to combat the spread of the COVID-19 pandemic (Table 4). 14.06% of the respondents supported the preventive measures, while 82.19% strongly supported the preventive measures. In terms of the effectiveness of preventive measures, 51.83% of the respondents strongly agreed that the precautionary measures could effectively prevent infection. Furthermore, a vast majority of the interviewees strongly agreed that citizens were obliged to take preventive measures during the epidemic, accounting for 72.67% of the total sample.

**Table 4:** Public attitudes toward preventive measures.

		**Beginning period, n (%)**	**Regular epidemic prevention and control period, n (%)**	**Total**	**χ^2^**	***p***
Support for preventive measures	Strongly Disagree	1 (0.08)	3 (0.27)	4 (0.17)	34.572	0.000^***^
	Disagree	4 (0.31)	8 (0.73)	12 (0.51)		
	Neutral	22 (1.72)	51 (4.66)	73 (3.07)		
	Agree	152 (11.87)	182 (16.64)	334 (14.06)		
	Strongly agree	1,102 (86.03)	850 (77.70)	1,952 (82.19)		
	Total	1,281	1,094	2,375		
Preventive measures can effectively avoid infection	Strongly Disagree	2 (0.16)	2 (0.18)	4 (0.17)	28.882	0.000^***^
	Disagree	6 (0.47)	15 (1.37)	21 (0.88)		
	Neutral	61 (4.76)	74 (6.76)	135 (5.68)		
	Agree	488 (38.10)	496 (45.34)	984 (41.43)		
	Strongly agree	724 (56.52)	507 (46.34)	1,231 (51.83)		
	Total	1,281	1,094	2,375		
It is a citizen's duty to take preventive measures	Strongly disagree	3 (0.23)	3 (0.27)	6 (0.25)	10.547	0.032^**^
	Disagree	4 (0.31)	11 (1.01)	15 (0.63)		
	Neutral	28 (2.19)	41 (3.75)	69 (2.91)		
	Agree	296 (23.11)	263 (24.04)	559 (23.54)		
	Strongly Agree	950 (74.16)	776 (70.93)	1,726 (72.67)		
	Total	1,281	1,094	2,375		

^***^p < 0.001, ^**^p < 0.01, ^*^p < 0.05.

Comparing public attitudes between the two periods shows that public attitudes toward preventive measures were better at the beginning of the epidemic than during the regular epidemic prevention and control period. Although most of the public believed that preventive measures were needed and had some confidence in their effectiveness, public attitudes toward preventive measures tended to decline with the recurrence of the epidemic.

#### Respondent's perception toward risk

Table 5 shows the public generally believes that COVID-19 is highly contagious and has a high mortality rate. 64.93% of respondents thought COVID-19 was very infectious; 33.05% of respondents believed the death rate of COVID-19 were relatively high; It can be seen that most respondents have some anxiety and fear about COVID-19. However, 52.04% of respondents believed that taking protective measures (such as wearing masks) is somewhat likely to catch COVID-19. It can be found that the public has some confidence in the recommended preventive measures.

**Table 5:** Public perception of risk.

		**Beginning period, *n* (%)**	**Regular epidemic prevention and control period, n (%)**	**Total**	**χ^2^**	***p***
How likely do you think you are to get COVID-19 if you go out with a mask?	Not at all likely	183 (14.29)	69 (6.31)	252 (10.61)	100.497	0.000^***^
	Less likely	722 (56.36)	514 (46.98)	1,236 (52.04)		
	Likely	264 (20.61)	322 (29.43)	586 (24.67)		
	Very likely	69 (5.39)	134 (12.25)	203 (8.55)		
	Almost certain	43 (3.36)	55 (5.03)	98 (4.13)		
	Total	1,281	1,094	2,375		
How likely do you think you are to get COVID-19 if you receive a courier from a region with a severe outbreak?	Not at all likely	328 (25.60)	19 (1.74)	347 (14.61)	693.488	0.000^***^
	Less likely	620 (48.40)	254 (23.22)	874 (36.80)		
	Likely	257 (20.06)	394 (36.01)	651 (27.41)		
	Very likely	58 (4.53)	362 (33.09)	420 (17.68)		
	Almost certain	18 (1.41)	65 (5.94)	83 (3.49)		
	Total	1,281	1,094	2,375		
How likely do you think COVID-19 is to be contagious?	Not at all likely	2 (0.16)	3 (0.27)	5 (0.21)	12.865	0.012^**^
	Less likely	7 (0.55)	14 (1.28)	21 (0.88)		
	Likely	24 (1.87)	42 (3.84)	66 (2.78)		
	Very likely	410 (32.01)	331 (30.26)	741 (31.20)		
	Almost certain	838 (65.42)	704 (64.35)	1,542 (64.93)		
	Total	1,281	1,094	2,375		
How likely do you think it is that you will die from getting COVID-19?	Not at all likely	46 (3.59)	61 (5.58)	107 (4.51)	12.731	0.013^**^
	Less likely	303 (23.65)	252 (23.03)	555 (23.37)		
	Likely	365 (28.49)	353 (32.27)	718 (30.23)		
	Very likely	441 (34.43)	344 (31.44)	785 (33.05)		
	Almost certain	126 (9.84)	84 (7.68)	210 (8.84)		
	Total	1,281	1,094	2,375		

^***^p < 0.001, ^**^p < 0.01, ^*^p < 0.05.

Comparing the public's risk perceptions between the two periods shows that the public's fear of the epidemic decreases and their fear of the disease diminishes as time passes. More people view the epidemic with a typical attitude.

#### Respondents' adoption of preventive actions

From the survey data, the survey respondents generally adopted a higher degree of preventive measures for the COVID-19 pandemic, which is related to the public knowledge on the preventive measures mentioned above and the supporting attitude toward the preventive measures (Table 6). Compared with other preventive measures, the number of respondents who chose to wear masks' frequency was higher. This situation is related to the need to wear a mask in public places during the epidemic.

**Table 6:** Public adoption of preventive actions.

		**Beginning period, *n* (%)**	**Regular epidemic prevention and control period, *n* (%)**	**Total**	**χ^2^**	***p***
Disinfect in time	Impossible	5 (0.39)	3 (0.27)	8 (0.34)	50.466	0.000^***^
	A bit possible	44 (3.43)	18 (1.65)	62 (2.61)		
	Possible	140 (10.93)	110 (10.05)	250 (10.53)		
	Very likely	497 (38.80)	576 (52.65)	1,073 (45.18)		
	For sure	595 (46.45)	387 (35.37)	982 (41.35)		
	Total	1,281	1,094	2,375		
Reduce going out	Impossible	19 (1.48)	19 (1.74)	38 (1.60)	69.84	0.000^***^
	A bit possible	56 (4.37)	63 (5.76)	119 (5.01)		
	Possible	116 (9.06)	149 (13.62)	265 (11.16)		
	Very likely	323 (25.21)	395 (36.11)	718 (30.23)		
	For sure	767 (59.88)	468 (42.78)	1,235 (52.00)		
	Total	1,281	1,094	2,375		
Wear masks	Impossible	5 (0.39)	4 (0.37)	9 (0.38)	275.034	0.000^***^
	A bit possible	41 (3.20)	13 (1.19)	54 (2.27)		
	Possible	194 (15.14)	63 (5.76)	257 (10.82)		
	Very likely	535 (41.76)	212 (19.38)	747 (31.45)		
	For sure	506 (39.50)	802 (73.31)	1,308 (55.07)		
	Total	1,281	1,094	2,375		
Daily body temperature	Impossible	11 (0.86)	6 (0.55)	17 (0.72)	59.573	0.000^***^
	A bit possible	58 (4.53)	58 (5.30)	116 (4.88)		
	Possible	222 (17.33)	200 (18.28)	422 (17.77)		
	Very likely	462 (36.07)	535 (48.90)	997 (41.98)		
	For sure	528 (41.22)	295 (26.97)	823 (34.65)		
	Total	1,281	1,094	2,375		

^***^p < 0.001, ^**^p < 0.01, ^*^p < 0.05.

The proportion of people wearing masks is significantly higher during regular epidemic prevention and control period. This is because the production of protective equipment such as masks reaches the demand during this period, there is no shortage of supply, and the public has access to the appropriate equipment.

### Test the hypothesized paths

Table 7 shows the results of the logit model. First, it can be seen from the first column that the research subjects have a high cognition of the COVID-19 virus and that, positive attitude is among the most critical factors influencing the adoption of personal protective behavior. These data confirm that the influencing factors; H1 and H2 are established, that is: interviewees who have a higher level of awareness of the COVID-19 virus and a positive attitude toward preventive measures are more inclined to adopt preventive behavior. The level of public knowledge is one of the crucial factors that affect behavior. When the public has a higher awareness of the disease, adopting prevention behavior will be more likely. The correctness and timeliness of its preventive behavior will be higher. In terms of attitude, when the public takes a positive attitude toward preventive measures, it means that they are more confident on the possibility of taking protective actions to reduce risks, and the actual adoption of activities will increase. However, the effect on the cognitive level was not significant during the regular epidemic prevention and control period, which may be related to the fact that as time progressed and the epidemic eased, people were less concerned about COVID-19 itself.

**Table 7:** Logit model results.

	**Total**	**Outbreak period**	**Regular epidemic prevention and control period**
Cognitive level	0.093^*^	0.127^**^	0.058
	(0.048)	(0.063)	(0.077)
Attitude	0.702^***^	0.661^***^	0.775^***^
	(0.115)	(0.183)	(0.154)
Information discrimination	0.714^***^	0.972^***^	0.515^***^
	(0.072)	(0.111)	(0.097)
Risk perception	0.307^***^	0.401^***^	0.18
	(0.088)	(0.123)	(0.127)
Dangerous attributes	0.381^***^	0.396^***^	0.454^***^
	(0.092)	(0.152)	0.123
Resource attributes	−0.074	−0.037	−0.062
	(0.056)	(0.082)	(0.08)
Stakeholder understanding	0.639^***^	0.716^***^	0.555^***^
	(0.097)	(0.129)	(0.151)
Stakeholder responsibility	0.183^**^	0.075	0.327^***^
	(0.069)	(0.091)	(0.108)
Education level	0.270^***^	0.288^***^	−0.111
	(0.073)	(0.085)	(0.164)
Income	0.195^***^	0.177^***^	0.134^**^
	(0.035)	(0.047)	(0.059)
Regional risk	0.003	−0.041	0.193
	(0.041)	(0.049)	(0.101)
Living location	0.211	0.088	0.489^*^
	(0.207)	(0.386)	(0.256)
Experience	−0.142	−0.152	−0.135
	(0.167)	(0.229)	(0.252)
Likelihood ratio test	χ^2^ (13) = 571.565, *p* = 0.000	χ^2^ (13) = 328.451, *p* = 0.000	χ^2^ (13) = 276.440, *p* = 0.000
*N*	2,375	1,281	1,094

^***^p < 0.001, ^**^p < 0.01, ^*^p < 0.05.

Secondly, the third-row results support H3, which hypothesized that; information resolution ability would significantly positively impact the willingness to prevent adoption. The regression coefficient value of the information resolution ability was 0.714 at a significance level of 0.01. The public with a stronger ability to distinguish information will have a better understanding of preventive behaviors and the higher the accuracy and timeliness of taking preventive practices, especially during the beginning period.

It can be seen from the results in Table 7 that the level of risk perception is positively correlated with the probability of the public adopting preventive behaviors. So H4 is established, and the public members with a higher awareness level of risk tend to have a stronger willingness to adopt protective actions. Their fear of the disease diminishes as time passes. More people view the epidemic with a typical attitude. The positive effect of the level of risk perception on the probability of the public adopting preventive behavior during the regular epidemic prevention and control period is not significant, possibly because their fear of the disease diminishes as time passes. More people view the epidemic with a typical attitude.

Among the two attributes of protective behavior perception, the regression coefficient value of the hazard attribute was 0.381, and at a 0.01 level of significance, showing that the hazard attribute will have a significant positive impact on the willingness to adopt preventive actions. The regression coefficient value of the resource attribute was −0.074. Still, it does not show significance, which means that the resource attribute does not affect the willingness to adopt preventive behaviors. In the early stage of the pandemic, there could have been resource insufficiencies. However, the Chinese government's strong execution force mobilized medical personnel and various protective resource production enterprises to increase production to ensure that most public can easily access the various protective equipment. Local governments have adopted various measures to combat the pandemic, resulting in weaker resource attributes.

In terms of stakeholder perception, stakeholder understanding will significantly impact the willingness to adopt preventive behaviors whereas stakeholder responsibility will not affect the desire to adopt during the beginning period.

At the same time, different demographic characteristics have different effects on the adoption of preventive measures. The education level and income level had a significant positive impact on the willingness to adopt preventive behaviors. Respondents with higher education levels tended to take preventive actions, and respondents with higher incomes are more capable of purchasing protective equipment to complete preventive practices. However, the effect of education level was not significant in times of regular epidemics, but rather the effect of exposure to confirmed patients was more pronounced.

## Discussions and implications

This study is essential in understanding the factors that affect the public's acceptance of recommended preventive measures during the different periods. From the survey data, the survey respondents generally adopted a higher degree of preventive measures of COVID-19. The degree of understanding, attitudes to preventive measures, ability to confirm the authenticity and truthfulness of information, risk perception, stakeholder understanding of the pandemic situation, risk attributes, education level, and salary level will significantly impact the adoption of preventive measures. Furthermore, it is of great significance to the relevant departments in providing references for related disease prevention and intervention strategies. The level of knowledge, attitudes to preventive measures, risk perceptions, and adoption behavior of respondents at the beginning of the epidemic were significantly higher than during the normalization. Over time, public concern and fear of the epidemic declined, and more people viewed the epidemic as usual. It is, therefore, necessary to tailor epidemic preparedness measures to different periods.

There is a link between information and knowledge dissemination and behavioral compliance. During a pandemic, many people cannot realize the impact compliance with the appropriate recommended behaviors can have on outbreak prevention or stopping the spread of an epidemic because they do not have sufficient knowledge or the right information. More importantly, in the current age of information explosion, people are often misinformed by false news or misinformation. Decision-makers must ensure three key characteristics: information quality, timeliness, and trustworthiness to increase public acceptance of the proposed measures. The relevant authorities need to report the occurrence and progress of an event openly and transparently and follow up continuously after the event to enhance the level of information perception to stabilize the public's response to the event. Corresponding information dissemination methods should be formulated for different groups to improve information dissemination effectiveness. For example, for people who do not frequently use the Internet and other news media, traditional media's propaganda efforts, such as television, newspapers, and radio, can be strengthened. Relevant departments need to strengthen education's role to improve information discrimination ability for people with low educational background. The means of disseminating health knowledge should be diversified. For example, posters, folders, and cartoons can attract the public and arouse their attention and interest in health knowledge or stimulate public participation in health knowledge activities through knowledge competitions, science talks, and skills competitions.

Secondly, risk perception plays a crucial role in predicting behavioral intentions. When people realize a strong correlation between risk perception and intent to act, they tend to follow the provided recommendations. Therefore, strengthening the communication ability between the government and the public by updating the risk status in time will help the public take corresponding preventive measures and reduce infectious diseases.

Thirdly, Stakeholders' understanding of the pandemic situation has a significant positive impact on adopting preventive measures by the public. The government needs to make the information available more transparently and openly. To enhance public trust in the government, the health department leaders need to strengthen their professional qualities to avoid shortcomings that are difficult to deal with in public health emergencies. At the same time, attention should also be directed to grass-roots managers' supervisory role, strengthen residents' self-detection and prevention, and ensure that infected persons are put on isolation for treatment in time.

Fourthly, risk attributes have a significant positive impact on the adoption of preventive measures. Relevant departments need to conduct a more comprehensive and detailed interpretation, introducing disease-related risk factors and prevention and control methods to strengthen public confidence in recommending preventive measures.

Fifthly, the general public needs to enhance their awareness of self-protection, develop good hygiene habits, wash hands regularly, frequently ventilate, wear masks, and frequently disinfect, etc. It is also necessary to enhance personal protection by strengthening physical fitness and improving personal immunity, maintaining a healthy lifestyle, eating a healthy diet to provide adequate nutrition, exercising in moderation to improve body resistance, and regularly working to maintain a good night's sleep.

In addition, when an epidemic lasts for a long time, 'pandemic fatigue' may set in and make people less willing to follow recommended behaviors. The level of knowledge, attitudes to preventive measures, risk perceptions, and adoption behavior of respondents at the beginning of the epidemic were significantly higher than during the normalization. When the epidemic subsides, the authorities should take measures to prevent people from letting their vigilance down. Government agencies should strengthen their monitoring role in such cases to promote behavioral change. During the regular epidemic prevention and control period, more emphasis should be placed on promoting prevention and control knowledge through diverse methods such as integrating new and traditional media to strengthen public awareness of epidemic prevention and to guard against prevention burnout. The relevant authorities need to update information throughout the event cycle according to the different dynamics and levels of disruption. At the same time, government agencies should strengthen their oversight role to promote behavioral change while ensuring the supply of protective materials and simplifying the management of the epidemic prevention and control process to reduce the cost of prevention and control behaviors to reduce “pandemic fatigue”.

## Conclusions

This study is essential in understanding the factors that affect the public's acceptance of recommended preventive measures during the different period. Furthermore, it is of great significance to the relevant departments in providing references for related disease prevention and intervention strategies. The level of knowledge, attitudes to preventive measures, risk perceptions and adoption behavior of respondents at the beginning of the epidemic were significantly higher than during the normalization of the epidemic. Over time, public concern and fear of the epidemic declined, and more people viewed the epidemic in a usual way. It is, therefore, necessary to tailor epidemic preparedness measures to different periods.

Using an online questionnaire for data collection means that respondents with only an internet connection are more likely to participate, which may lead to errors, such as a disproportionate number of well-educated people in this survey. Therefore, the findings may not be representative of the views of less-educated people. Also, the small sample size obtained for this survey in areas with severe outbreaks limits the derivation of conclusions. Subsequently, further research can be carried out by expanding the sample size and improving the sampling method.

The design of the COVID-19 cognitive level scale was based on the COVID-19 Prevention and Control Programme and the Public Protection Guidelines, which may need to be more comprehensive. While residents' behavior in complying with epidemic prevention policies and systems is studied from a holistic perspective, there may be differences in residents' psychological perceptions and actual behavior toward mandatory and non-mandatory requirements. Future studies can examine different types of epidemic prevention policies and measures separately or in comparison to enrich the findings on residents' adoption of epidemic prevention behavior.

## Data availability statement

The raw data supporting the conclusions of this article will be made available by the authors, without undue reservation.

## Author contributions

FF: conceptualization, methodology, and writing—review and editing. SC: formal analysis and writing—original draft preparation. XG and EK: writing—review and editing. All authors read and approved the final manuscript.
